# Changes in Metabolic Hormones in Malaysian Young Adults following *Helicobacter pylori* Eradication

**DOI:** 10.1371/journal.pone.0135771

**Published:** 2015-08-20

**Authors:** Theresa Wan-Chen Yap, Alex Hwong-Ruey Leow, Ahmad Najib Azmi, Fritz Francois, Guillermo I Perez-Perez, Martin J. Blaser, Bee-Hoon Poh, Mun-Fai Loke, Khean-Lee Goh, Jamuna Vadivelu

**Affiliations:** 1 Department of Medical Microbiology, Faculty of Medicine, University of Malaya, 50603, Kuala Lumpur, Malaysia; 2 Department of Medicine, Faculty of Medicine, University of Malaya, 50603, Kuala Lumpur, Malaysia; 3 New York University Cancer Institute, New York University School of Medicine, New York, New York, United States of America; 4 Department of Medicine, New York University School of Medicine, New York, New York, United States of America; 5 Department of Microbiology, New York University School of Medicine, New York, New York, United States of America; 6 BP Healthcare, 30250 Ipoh, Perak, Malaysia; McMaster University, CANADA

## Abstract

**Background:**

More than half of the world’s adults carry *Helicobacter pylori*. The eradication of *H*. *pylori* may affect the regulation of human metabolic hormones. The aim of this study was to evaluate the effect of *H*. *pylori* eradication on meal-associated changes in appetite-controlled insulinotropic and digestive hormones, and to assess post-eradication changes in body mass index as part of a currently on-going multicentre ESSAY (Eradication Study in Stable Adults/Youths) study.

**Methods:**

We enrolled 29 *H*. *pylori*-positive young adult (18–30 year-old) volunteer subjects to evaluate the effect of *H*. *pylori* eradication on meal-associated changes on eight gastrointestinal hormones, using a multiplex bead assay. Changes in body mass index and anthropometric measurements were recorded, pre- and post-eradication therapy.

**Results:**

Pre-prandial active amylin, total peptide YY (PYY) and pancreatic polypeptide (PP) levels were significantly elevated 12 months post-eradication compared with baseline (n = 18; Wilcoxon's signed rank test, p<0.05). Four of the post-prandial gut metabolic hormones levels (GLP-1, total PYY, active amylin, PP) were significantly higher 12 months post-eradication compared to baseline (n = 18; p<0.05). Following *H*. *pylori* eradication, the BMI and anthropometric values did not significantly change.

**Conclusions:**

Our study indicates that *H*. *pylori* eradication was associated with long-term disturbance in three hormones (active amylin, PP and total PYY) both pre- and post-prandially and one hormone (GLP-1) post-prandially. Longer post-eradication monitoring is needed to investigate the long-term impact of the observed hormonal changes on metabolic homeostasis.

## Introduction

*Helicobacter pylori* is a Gram negative, spiral-shaped, microaerophilic bacterium that colonizes the gastric mucosa of humans and non-primates. More than half of the world’s adults carry *H*. *pylori* and its prevalence is as high as 80% in some populations [1]. Individuals usually acquire *H*. *pylori* in early childhood and remain asymptomatic during most of their lifetime but some may develop symptoms later in life [2]. Carriage of *H*. *pylori* is involved in the development of duodenal and gastric ulcers, and is also strongly associated with the development of two gastric malignancies; mucosa-associated lymphoid tissue (MALT) lymphoma and gastric adenocarcinoma, and has been classified as a group I carcinogen [3].

On the other hand, *H*. *pylori* is part of the human endogenous microbiome; recent studies provide evidence that it has been present in the human stomach for at least 100,000 years [4]. To ensure their survival in the gastric environment, *H*. *pylori* has acquired traits of evading and subverting both the innate and adaptive immune systems during the long period of co-evolution [5]. However, due to socio-economic development, modern hygienic practices and the advent of antibiotics, there is growing evidence that the human gastrointestinal tract (GI) microbiota has changed, and that *H*. *pylori* is gradually disappearing [6].

Differences in the GI microbiome of obese and lean individuals may affect their metabolic potential, which may confer greater capacity to harvest energy, contributing to obesity [7]. Therefore, it is possible that the changes in the GI microbiome, especially involving the major gastric bacterium *H*. *pylori*, are associated with changes in the regulation of metabolic hormones and consequently, contribute to obesity. Several epidemiological and experimental studies support a protective effect of *H*. *pylori* against the development of obesity [8–10].

Leptin and ghrelin are two centrally-acting hormones that have a major influence on energy homeostasis in mammals [11]. Leptin, produced mainly by adipose tissue, is a long-term mediator of energy balance. It may play a role in reducing appetite, and increasing energy utilization. In contrast, ghrelin is a fast-acting hormone mainly secreted by the stomach that may to play a role in feeding behavior and decreased energy expenditure [8, 11]. Ghrelin serum levels are high before meals and decrease post-prandially [12, 13]. Although it has been hypothesized that leptin and ghrelin have opposing effects on human energy homeostasis [11, 14], several studies have produced conflicting results [13, 15–17].

We hypothesize that the eradication of *H*. *pylori* may affect the regulation of human metabolic hormones involved in food consumption, energy homeostasis, and body weight maintenance. Acyl-ghrelin and leptin, which are produced in the stomach and adipose tissue, respectively, as well as active amylin, insulin, glucagon-like peptide-1 (GLP-1), total gastric inhibitory polypeptide (GIP), total peptide YY (PYY), and pancreatic polypeptide (PP), all produced in the GI tract distal to the stomach, were assessed. Clinically indicated *H*. *pylori* eradication was used to evaluate the effect of eradication on meal-associated changes in these appetite-controlled insulinotropic or digestive hormones, and to assess post-eradication changes in body mass index, as described [8].

## Methods

### Study population and Clinical evaluation

This study is part of the on-going ESSAY (Eradication Study in Stable Adults/Youths) study in Kuala Lumpur and New York (New York University Langone Medical Center). In Kuala Lumpur, it was conducted at the University of Malaya Medical Centre (UMMC), Malaysia. Malaysian young adult volunteers (age range from 18 to 30 years) were consecutively recruited between June 2012 and November 2013, and were screened to assess eligibility for the study based on a priori exclusion criteria, including diabetes, hyper or hypothyroidism, prior gastric or bariatric surgery, prior documented *H*. *pylori* treatment, antibiotic, steroid or other immunomodulating drug use within 4 weeks of enrollment, recent vaccination, and Charlson weighed comorbidity index <2. Demographic information collected via a baseline questionnaire, included ethnic designation (self-reported as Malay, Chinese, Indian or Other). Height and weight were measured and body mass index (BMI) was calculated. The study protocol was reviewed and approved by the UMMC Medical Ethics Committee (Ref No. 877.1). Written informed consent was obtained from qualified candidates prior to study participation. Fifteen ml of blood was collected, centrifuged, and stored as serum at -80°C until examined. Urea Breath Test (UBT) and *H*. *pylori* serology were performed to determine *H*. *pylori* status of the volunteers.

### ^13^C-Urea Breath Test

*H*. *pylori* testing was performed using the non-radioactive ^13^C-UBT (Isotope-Selective Infrared Spectroscopy method), shown to have a sensitivity of 91% and a specificity of 93% in our local population [18]. The technique involves the consumption of 75 mg of a ^13^C-urea solution after collection of a baseline breath sample. Breath samples then were collected 10, 20 and 30 min post ^13^C-urea administration, and the concentrations of isotope-labeled carbon dioxide analyzed using an IRIS infrared isotope analyzer (Wagner Analysen Technik, Bremen, Germany). A delta-over-baseline value (DOB) >4% indicates *H*. *pylori* positivity.

### *H*. *pylori* serology

Anti-*Helicobacter pylori* IgA & IgG antibodies in serum were determined quantitatively using the Pyloriset EIA-A III & EIA-G III (Orion Diagnostica, Espoo, Finland) [19, 20]. Result interpretation was as follows:

Positive ≥20 U/ml; Negative <20 U/ml. Volunteers were considered as *H*. *pylori*-positive, if UBT and serology tests were both positive.

### Endoscopy

Each volunteer fasted for 12 hours overnight prior to endoscopy. After intravenous administration of meperidine and midazolam, complete endoscopic evaluation of the upper gastrointestinal tract was performed in standard fashion to the second portion of the duodenum, as described [21]. Gastric inflammation was graded using the Sydney-Houston system [22]. Using standard forceps, two biopsies were obtained from the gastric antrum for histopathological analysis, two for rapid urease testing (RUT), and two for *H*. *pylori* culture. Positivity by RUT, histological examination, and culture were considered as confirmatory for *H*. *pylori* positivity.

### Histological analysis

Biopsy specimens from the antrum were fixed in 10% formalin, embedded in paraffin, sectioned, and hematoxylin and eosin (H & E) stained [23]. A single experienced GI pathologist, blinded to the data, graded the extent of gastritis based on the presence or absence of *H*. *pylori*, chronic inflammation (mononuclear cells), polymorphonuclear neutrophil activity, glandular atrophy, and intestinal metaplasia based on a *mild*, *moderate* or *marked* scale, according to the updated Sydney classification [24]. The presence of polymorphonuclear neutrophil activity indicates active gastritis while presence of both polymorphonuclear neutrophil activity and mononuclear cells indicates chronic active gastritis [24]. *H*. *pylori* was detected as spiral or curved organisms near the mucous layer.

### *H*. *pylori* culture

Gastric biopsy samples obtained from the antrum of the stomach were homogenized and plated onto non-selective and selective chocolate agar supplemented with 7% lysed horse blood (Oxoid, Hampshire, UK). Selective chocolate agar contained vancomycin (10 μg/ml), amphotericin B (5 μg/ml), trimethoprim (5 μg/ml) and nalidixic acid (20 μg/ml) [25]. The inoculated agar plates were incubated for 3–10 days in a humidified 10% CO_2_ incubator at 37°C.

### Baseline visit

*H*. *pylori*-positive volunteers were instructed to arrive at the Endoscopic Unit of UMMC at 8am after 12 hours of fasting. Anthropometric measurements (height, weight, waist & hip circumference, and triceps-skinfold) were obtained. Validated Severity of Dyspepsia Assessment questionnaire (SODA) and SF-36 Health Survey were administrated to volunteers to assess baseline symptoms and quality of life profile.

Five ml of fasting blood was collected in EDTA-coated tube. The protease inhibitor, 4-(2-Aminoethyl) benzenesulfonyl fluoride hydrochloride (AEBSF) (Sigma-Aldrich, St. Louis MO), was added to the blood collection tubes at a concentration of 1 mg/ml. Tubes were kept on ice until centrifuged, and plasma was frozen at -80°C for metabolic hormone analysis.

### Test meal

Following the fasting blood draw, volunteers consumed a standard 16-oz liquid meal totalling 700 calories (2 cans of Ensure Plus) (Abbott Laboratories, Abbott Park IL). The contents of the meal provide 100 g carbohydrate, 26 g protein, and 22 g fat (as reported by the manufacturer). Since it has been reported that serum ghrelin levels change one hour post-prandially [13], 5 ml of blood was collected at that time.

### *H*. *pylori* eradication therapy

Treatment with a 7-day twice daily regimen and a proton pump inhibitor as per current standard of care (amoxicillin 1000 mg, clarithromycin 500 mg, and pantoprazole 40 mg) were offered to volunteers who tested positive for *H*. *pylori*. *H*. *pylori* eradication was ascertained using the non-radioactive ^13^C Urea Breath Test, ≥ 6 weeks after completion of the treatment protocol. Volunteers who failed the first-line eradication regime were given second-line eradication therapy with a 2-week twice daily regime (amoxicillin 1000 mg, levofloxacin 500 mg and rabeprazole 20 mg).

### Follow-up assessment

Volunteers returned for follow-up assessment at 6 and 12 months, paralleling that described for the baseline visit.

### Metabolic hormone testing

MILLIPLEX MAP Kit-Human Metabolic Hormone Magnetic Bead Panel (EMD Millipore, Billerica MA) was used to quantify gut hormones that are important regulators of food intake, energy expenditure, and body weight via the gut-brain axis in plasma. These include acyl-ghrelin (active), leptin, active amylin, insulin, GLP-1, total GIP, total PYY, and PP. Measurement was performed on Luminex 200 system with xPONENT software, version 3.1 (Luminex, Austin TX).

### Statistical analysis

Depending on their distribution, data were expressed as mean ± SD, or median and 95% confidence interval (CI). Continuous variables were compared using the *t*-test, or one-way ANOVA, and pairwise analyses (e.g. pre-prandial vs. post-prandial, baseline vs. 6 months post-eradication, baseline vs. 12 months post-eradication) were performed using non-parametric tests (Wilcoxon's signed rank test, Mann-Whitney U test), as appropriate. Statistical analysis was performed using SPSS software version 20.0 (SPSS Inc., Chicago IL); a two-tailed p-value of <0.05 was considered significant [8].

## Results

### Subject demographic and Clinical analysis

**Fig 1:**
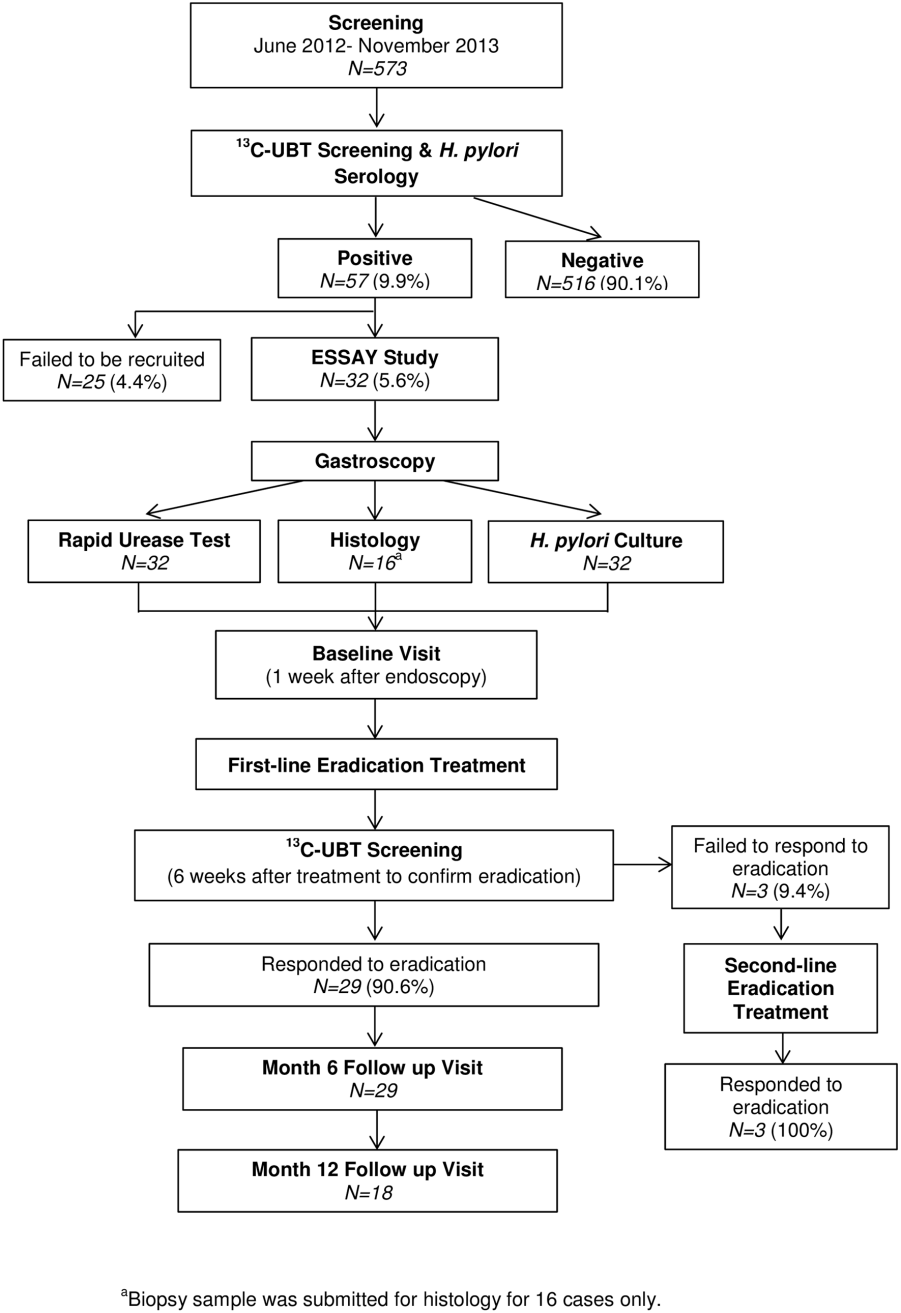
Summary of ESSAY Study.

Summary of the recruitment was shown in Fig 1. Initially, 573 healthy adult volunteers participated in our study. Of the 573 volunteers recruited, 67 (11.7%) tested positive using UBT. The volunteers were also screened for *H*. *pylori* serology in which a large fraction was found to be IgA, IgG, or both IgA/IgG positive, respectively (S1 Table). Of the 573 volunteers screened in this study, 57 (9.9%) tested positive using both UBT and detection of anti-*H*. *pylori* antibodies and were considered as *H*. *pylori*-positive. However, only 32 (5.6%) consented to participate in the ESSAY Study (S1 Table). Of these 32 volunteers, only 16 of them consented to undergo gastroscopy and biopsy samples were obtained. Their *H*. *pylori* infection status was further confirmed using RUT, histological examination, and culture. Histological analysis showed the presence of both neutrophils and mononuclear cells in all the samples which indicated that these *H*. *pylori* positive volunteers all had chronic active gastritis (S2 Table). First-line eradication treatment was given to the 32 *H*. *pylori* positive volunteers but was successful for only 29 (90.6%). Twenty-nine of these volunteers were recruited into the ESSAY Study. For the remaining three (9.4%) volunteers who failed to respond, second-line eradication treatment was prescribed. They were excluded from this study, since their number was small and they differed from the primary eradication group.

The majority of the 29 volunteers eventually recruited into the ESSAY Study were medical students of the University of Malaya. The mean age of the volunteers was 24.9 years and 23 (79%) were female. The volunteers consisted of 5 (17%) Malay, 8 (28%) Chinese, 13 (45%) Indian while the remaining 3 (10%) came from other ethnicities. The mean BMI at baseline was 22.45 kg/m^2^ which was consistent with the normal weight category (World Health Organization) [26]. The 29 volunteers were followed up to 6 months post-*H*. *pylori* eradication. However, 11 volunteers dropped out of the study at 12 months post- *H*. *pylori* eradication. Thus, we could only manage to obtain follow-up to one year with 18 of them (Fig 1).

Assessing the quality of life profile and volunteers’ health condition, using the SF-36 Health Survey, showed that, the volunteers expressed that their health condition had improved 6 months post-eradication of *H*. *pylori*, compared to the previous year (Mann Whitney U-Test, p< 0.05). However, the survey also showed that both physical and emotional health of volunteers four weeks prior to the survey, (versus pre- and post-*H*. *pylori* eradication), had not affected their performance at work or other regular daily activities and did not interfere with their normal social activities (data not shown).

### Meal-associated changes of metabolic hormones and effects of *H*. *pylori* eradication

The levels of the eight studied gut metabolic hormones varied markedly among the subjects in both the pre-prandial and post-prandial sera (Table 1). The studied hormones responded to the test meal, as predicted [27–31]. Insulin, active amylin, total GIP, GLP-1 and PP levels rose physiologically and significantly (p<0.05) after the test meal, at all three time points (baseline, 6 months and 12 months post-*H*. *pylori* eradication). At baseline, post-prandial total PYY levels did not rise significantly, but did 6 months and 12 months after *H*. *pylori* eradication. At baseline and 12 months post-*H*. *pylori* eradication, but not at 6 months post-eradication, levels of acyl-ghrelin decreased significantly following the test meal. Leptin decreased significantly post-prandially at baseline and 6 months post-*H*. *pylori* eradication but not at 12 months post-eradication.

**Table 1:** Eight Metabolic Hormone Levels in Studied Subjects According to *H*. *pylori* Eradication Status.

Hormone	Median (95% CI*) hormone concentration (pg/ml)												
	Baseline (N = 29)			6 months post-eradication (N = 29)			12 months post-eradication (N = 18)			Comparison of hormone concentration at baseline and 6 months post-eradication (p-value)^a^		Comparison of hormone concentration at baseline and 12 months post-eradication (p-value)^a^	
	Pre-prandial	Post-prandial	p-value^b^	Pre-prandial	Post-prandial	p-value^b^	Pre-prandial	Post-prandial	p-value^b^	Pre-prandial	Post-prandial	Pre-prandial	Post-prandial
Active Amylin	7.3	44.3	**0.013**	18.5	46.1	**0.006**	37.8	98.6	**0.008**	0.24	0.48	**0.036**	**0.017**
	(0–31.1)	(34.2–52)		(0–24.9)	(31.1–53.6)		(0–84.1)	(54.8–136.4)					
Acyl-ghrelin	12.7	10.2	**0.022**	11.8	12	0.45	15.4	7.6	**0.022**	0.26	0.29	0.17	0.72
	(6.5–19.4)	(7.1–16.4)		(7.1–17.1)	(6.5–16.5)		(8.8–27.1)	(6.5–19.6)					
Total GIP	25.4	281.3	**0.005**	35.1	222.2	**0.002**	41.1	276	**0.005**	0.88	0.64	0.09	**0.022**
	(15.4–52.5)	(190.8–302.5)		(20.7–48.5)	(200.8–317.8)		(25.1–70.7)	(237–430.4)					
GLP-1	17.4	44.4	**0.013**	17.4	49.6	**0.002**	22.5	63.5	**0.005**	0.21	**0.041**	0.68	**0.005**
	(13.6–32.1)	(25.6–55.5)		(0–29.1)	(37–59.6)		(0–47.5)	(47.2–84.2)					
Insulin	293.3	2235.4	**0.003**	292.1	1937.9	**0.002**	504	3455.3	**0.005**	0.88	0.16	0.14	0.09
	(209.5–392.2)	(1842.5–3198.5)		(251.4–445.3)	(1518.5–2436.6)		(310–559.4)	(2252.8–4449.2)					
Leptin	11091.8	9510.6	**0.016**	10917.7	9805	**0.012**	6059.6	7947	0.17	0.42	0.16	0.86	0.44
	(7318.1–13249.7)	(6427.7–10939.4)		(5477.9–13279.9)	(5124.9–13090.1)		(3000.1–16842.4)	(5216.5–14507.5)					
PP	16.1	75.5	**0.004**	16.4	88.3	**0.002**	33	136.7	**0.005**	0.65	0.29	**0.028**	**0.009**
	(0–39.5)	(52.4–100.2)		(0–34)	(55.8–157.8)		(5.6–62.9)	(77.9–229.2)					
Total PYY	30.7	65.9	0.05	35.6	86.5	**0.002**	96.3	174.4	**0.007**	1	**0.006**	**0.021**	**0.005**
	(0–44.6)	(0–83.1)		(0–53.4)	(77.2–95.7)		(0–150.2)	(140.9–219.5)					

p-values< 0.05 were indicated in bold.

*Bootstrapped 95% confidence interval (CI) was based on 1000 replicates.

^a^Wilcoxon’s signed rank test was used in comparing baseline and post-*H*. *pylori* eradication.

^b^Wilcoxon’s signed rank test was used in comparing pre-prandial and post-prandial values.

The levels of the eight gut metabolic hormones did not differ significantly pre-prandially between baseline and 6 months post-eradication. However, when baseline was compared with the 12 months post-eradication values, pre-prandial active amylin, total PYY and PP levels were significantly elevated (p<0.05). Median pre-prandial active amylin was increased by 5-fold from 7.3 pg/ml increased to 37.8 pg/ml (Baseline 95% CI: 0–31.1 pg/ml vs. 12 Months 95% CI: 0–84.1 pg/ml). Median pre-prandial total PYY was increased by 3-fold from 30.7 pg/ml increased to 96.3 pg/ml (Baseline 95% CI: 0–44.6 pg/ml vs. 12 Months 95% CI: 0–150.2 pg/ml). Median pre-prandial PP was increased by 2-fold from 16.1 pg/ml elevated to 33 pg/ml (Baseline 95% CI: 0–39.5 pg/ml vs. 12 Months 95% CI: 5.6–62.9 pg/ml).

The post-prandial hormonal changes between baseline and 6 months post-eradication also did not show significant difference except for total PYY (Baseline, median = 65.9 pg/ml; 95% CI: 0–83.1 pg/ml vs. 6 Months, median = 86.5 pg/ml; 95% CI: 77.2–95.7 pg/ml), which was approximately 1.3-fold higher 6 months post-eradication than baseline (p<0.05). However, by 12 months post-eradication, post-prandial levels of four of the gut metabolic hormones (GLP-1, total PYY, active amylin, PP) were significantly higher than at baseline (p<0.05). Median post-prandial total PYY was increased by 2.6-fold (Baseline, median = 65.9 pg/ml; 95% CI: 0–83.1 pg/ml vs. 12 Months, median = 174.4 pg/ml; 95% CI: 140.9–219.5 pg/ml). Median post-prandial active amylin was increased by 2.2-fold (Baseline, median = 44.3 pg/ml; 95% CI: 34.2–52 pg/ml vs. 12 Months, median = 98.6 pg/ml; 95% CI: 54.8–136.4 pg/ml). Median post-prandial PP was increased by 1.8-fold (Baseline, median = 75.5 pg/ml; 95% CI: 52.4–100.2 pg/ml vs. 12 Months, median = 136.7 pg/ml; 95% CI: 77.9–229.2 pg/ml). Median post-prandial GLP-1 was increased by 1.4-fold (Baseline, median = 44.4 pg/ml; 95% CI: 25.6–55.5 pg/ml vs. 12 Months, median = 63.5 pg/ml; 95% CI: 47.2–84.2 pg/ml) (Table 1).

### Effects of *H*. *pylori* eradication on body mass index and anthropometry

Twelve months following eradication of *H*. *pylori*, the BMI of volunteers did not significantly change. Correspondingly, waist, hip, and triceps circumference values also did not change significantly. Triceps circumference was significantly increased 6 months post-*H*. *pylori* eradication (Table 2).

**Table 2:** Comparison of BMI, and Waist, Hip, and Triceps Circumference Values According to *H*. *pylori* Eradication Status.

Characteristic	Mean BMI (kg/m^2^)/Waist Circumference (cm)/Hip Circumference (cm)/Triceps Skinfold (cm)				
	Baseline (N = 29)	6 months post-eradication (N = 29)	12 months post-eradication (N = 29)	Comparison between baseline and 6 months post-eradication (p-value)^a^	Comparison between baseline and 12 months post-eradication (p-value)^a^
**BMI**	22.45	22.75	23.55	0.093	0.061
**Waist Circumference**	76.67	74.56	75.35	0.209	0.130
**Hip Circumference**	94.83	94.81	97.96	0.992	0.831
**Triceps Skinfold**	1.57	1.89	1.98	**0.024**	0.059

p-values< 0.05 were indicated in bold.

^a^Paired t-test was used to compare BMI, and waist, hip, and triceps circumference values at baseline to post-eradication.

### Dyspepsia symptoms pre- and post-eradication of *H*. *pylori*

The Severity of Dyspepsia Assessment (SODA) questionnaire was given to the volunteers at baseline and at the follow-up visits to assess dyspeptic symptoms following *H*. *pylori* eradication. The SODA questionnaire demonstrated that the intensity of abdominal discomfort (or stomach-ache) 7 days prior to the survey, on average, had dropped significantly (Mann Whitney U-Test, p< 0.05) comparing baseline to post-eradication visits. Following *H*. *pylori* eradication, the satisfaction level of volunteers towards the current level of abdominal discomfort (or stomach-ache) due to dyspepsia had increased significantly (Mann Whitney U-Test, p< 0.05). Other signs and symptoms associated with dyspepsia (bad breath, burping/belching, heartburn, bloating, passing gas, sour taste, and nausea) did not significantly present problems to the volunteers before or after *H*. *pylori* eradication (data not shown).

## Discussion

This study focused on a young and healthy Malaysian population with low risk of diseases, based on the premise that they have normal and balanced energy homeostasis, making them an excellent target population for investigating the effect of *H*. *pylori* eradication on gastrointestinal hormones in relation to metabolism.

Ethnic differences in the prevalence of *H*. *pylori* in this region have been reported. In Malaysia, the prevalence of *H*. *pylori* infection of Indians (29%-62%) is consistently higher than in Chinese (18%-58%) and Malays (7%-29%) [32–37]. Hence, the racial distribution of volunteers recruited into this study reflects the *H*. *pylori* prevalence among the various ethnic groups in Malaysia. Analysis of seven housekeeping genes by multilocus sequence typing (MLST) has shown that *H*. *pylori* hpAsia2/hspIndia sub-population predominates among Indians and Malays while hpEastAsia/hspEAsia sub-population predominates among Chinese of Malaysia [38]. In addition, prevalence of the cytotoxin-associated gene A (*cagA*), among *H*. *pylori* clinical isolates was reported to be 86%, 95%, and 94% in Malays, Chinese, and Indians, respectively [39]. As for the vacuolating cytotoxin A (*vacA*) gene, the prevalence of *vacA* m1 genotype was similar among Malays (64%), Chinese (55%), and Indians (70%). The prevalence of *vacA* s1 and i1 genotypes among different races were reported to be as high as 90%-100% [39]. Therefore, the prevalence of *cagA* and *vacA* genes are similar among different ethnic groups in Malaysia.

According to the SF-36 Healthy Survey, the *H*. *pylori*-positive volunteers who participated in this study and completed the test meal and follow-up visit were healthy and fit physically and emotionally. *H*. *pylori* eradication did not affect their physical or emotional health, and their personal perception of their health status, but in this unblinded study, it was associated with decreased dyspeptic symptoms. Because of important placebo effects in clinical trials of dyspepsia [40, 41], the decrease in symptoms should be interpreted with caution.

Normal energy homeostasis in humans is controlled by signalling pathways that involve hormones (leptin and acyl-ghrelin) with prominent central nervous system activity and hormones (insulin, active amylin, incretins (total GIP, GLP-1), PP, and total PYY) that have major activity in the GI tract and in other peripheral tissues. Due to its intimate relationship with the gastric epithelium, *H*. *pylori*, the dominant member of the gastric microbiota, has the potential to affect the regulation of normal energy homeostasis of humans [8–10, 42–45].

Ghrelin is a circulating peptide that serves as one of the key regulators of energy homeostasis [11, 46]. Through multiple synthesis steps, ghrelin is acylated to form acyl-ghrelin, believed to be the most active form of ghrelin that induces positive energy balance [47]. The majority of human acyl-ghrelin is produced by X/A-like cells in the gastric oxyntic mucosa [48]. Ghrelin has substantial roles in energy homeostasis including meal initiation, adipogenesis, and body weight gain, mediated through the hypothalamus [8, 11, 46]. The secretion of ghrelin by the gastric mucosa depends largely on nutritional state and the levels of leptin [11]. In contrast, leptin is produced primarily by adipose tissue and has important hypothalamus-dependent roles in regulating food intake and energy expenditure [8, 11].

The effects of leptin and ghrelin on energy homeostasis may be opposing; leptin induces weight loss by suppression of food consumption, while ghrelin acts as an appetite-stimulatory signal [8, 11]. In our study, *H*. *pylori* eradication had a transient effect on acyl-ghrelin levels, with the loss of meal-induced suppression, but by 12 months, normal physiology was restored. In contrast, the regulation of leptin appeared to be disturbed 12 months post-*H*. *pylori* eradication. Under normal circumstances, the levels of leptin decreased post-prandially. However, leptin levels were stimulated in response to the standard meal 12 months post-*H*. *pylori* eradication. These data provided evidence that eradication of *H*. *pylori* may have long-term effects on both acyl-ghrelin and leptin levels. Furthermore, this could also suggest that inter-meal ghrelin levels may display a diurnal rhythm, that is in phase with that of leptin in healthy humans [13]. A recent study also reported that leptin administration to healthy volunteers did not regulate ghrelin levels over several days [15]. The results obtained in this study are consistent with prior findings, providing evidence that the circulating acyl-ghrelin level is not regulated by leptin, but instead both hormones may have independent roles in regulating energy homeostasis.

Whether the eradication of *H*. *pylori* is associated with increased or decreased levels of ghrelin remains a subject of controversy [8, 9, 49, 50]. In this study, *H*. *pylori* eradication had no significant effect on the levels of acyl-ghrelin, consistent with an earlier report that both pre-prandial and post-prandial plasma ghrelin levels remain unchanged after *H*. *pylori* eradication [9]. As with ghrelin, conflicting findings have been reported on the effect of *H*. *pylori* eradication on meal-associated leptin level [8, 49, 51, 52]. Similarly, both pre-prandial and post-prandial plasma leptin levels in this study remained unchanged post-*H*. *pylori* eradication.

Other studies found concomitant changes in ghrelin and leptin levels with change in BMI and a positive correlation between ghrelin, leptin and BMI in patients after *H*. *pylori* eradication [8, 52]. However, since our study showed that the BMI of volunteers did not significantly change following *H*. *pylori* eradication, correlation analysis could not be performed.

The contradicting results between metabolic and anthropometric measures across different studies may reflect methodological differences including variation in demographic characteristics (health status, gastrointestinal disease conditions, socio-economic status, age, gender, ethnicity), geographic settings (Western vs. Eastern populations), diet, lifestyle, and length of post-*H*. *pylori* eradication therapy follow-up. The extent and location of *H*. *pylori*-induced inflammation at baseline may be associated with differences in ghrelin physiology that develop with *H*. *pylori* eradication [8, 44, 53]. In one study, subjects with only antral gastritis showed the largest increases in ghrelin levels obtained pre- and post-prandial, and across the meal ghrelin levels [8]. Without biopsies from the gastric corpus, we could not determine inflammation status, and thus could not confirm or refute these findings.

In our study, as expected, gastrointestinal hormones (insulin, active amylin, incretins (total GIP, GLP-1), PP and total PYY) increased significantly after the consumption of the standard meal. Following a meal, the blood glucose level rises and insulin is released to increase glucose uptake [54]. Amylin, co-localized with insulin, is simultaneously secreted to assist with glycemic regulation by slowing gastric emptying and promoting satiety, thus minimizing post-prandial spikes in the blood glucose level [55, 56]. GIP and GLP-1, synthesized primarily by K cells at the mucosa of the duodenum and jejunum and ileal L-cells respectively, are incretins [27, 57]. Incretins promote insulin secretion [57], reduce the rate of nutrient absorption into the bloodstream and slow gastric emptying [55]. PP and PYY increase post-prandially and aid in metabolic regulation by regulating pancreatic secretion [28, 58]. These gut hormones also play important roles in energy homeostasis through their effects on neuroendocrine signalling within the brain [28, 55]. Through this intricate cross-regulated system, the gastrointestinal tract, adipose tissue, and brain can communicate efficiently in regulating energy intake.

*H*. *pylori* eradication was associated with long-term disturbances in gut hormones not secreted by the stomach, three (active amylin, PP and total PYY) both pre- and post-prandially and one hormone (GLP-1) only post-prandially. The mechanisms for these changes are unknown, but could be associated with perturbation of gastric hormones, including gastrin [59], or host immunity [60], and/or the gut microbiota compositions [61, 62] following *H*. *pylori*-eradication, that in turn might modulate the human gut hormones and metabolism. The fecal 16S metagenomics analysis of the same cohort of volunteers has shown that the relative abundance of short-chain fatty acid (SCFA)-producing bacteria was elevated post-*H*. *pylori* eradication (data not shown). Interestingly, SCFA also have been reported to stimulate the release of hormone PYY and GLP-1 in both rodent and human via activation of the G-protein-coupled receptor [63–67]. Therefore, we suggest that the antibiotic therapy used for *H*. *pylori* eradication may also cause perturbation of the gut microbiota, which is in turn may interfere with the regulation of human energy homeostasis.

The primary strength of our study was we were able to measure the pre- and post-prandial changes of the eight gut hormones in asymptomatic young adults to ascertain the metabolic profile of these volunteers at baseline and follow them up to 12 months post-*H*. *pylori* eradication. In addition, we also collected detailed demographics and clinical data as well as utilizing multiple methods for *H*. *pylori* status determination from a prospectively enrolled group consisting of 573 volunteers. One of the major limitations of our study was a high dropout rate (approximately 40%) of volunteers from 12 months post-eradication follow-up due to various reasons unrelated to the study. We also planned to recruit volunteers who failed the first-line eradication therapy, as well as *H*. *pylori*-negative volunteers in the control group; however few *H*. *pylori*-negative individuals recognized the benefit of joining the study and thus, both groups were not sufficiently populated to carry out a well-powered study. Diet may contribute partially to the relatively stable anthropometric measurements of the volunteers at baseline and post-*H*. *pylori* eradication. However, diet and calorie intake information were not included as part of the survey, which was another limitation of this present study.

In conclusion, our study indicates that eradication of *H*. *pylori* affects the regulation of human metabolic hormones involved in appetite-control and energy homeostasis, particularly the long term elevation of hormone PP and PYY could be associated with the perturbation of gut microbiome and eventually lead to the development of metabolic disorders. Longer monitoring of volunteers from the ESSAY cohort is necessary to investigate the impact of *H*. *pylori*-eradication-associated hormonal changes on weight maintenance and other physiologic measures.

## Supporting Information

S1 Table Summary of *H*. *pylori* Status Screening. (PDF)

S2 Table Summary of Histopathology Findings. (PDF)
