# Supplementary material for: Changes in Metabolic Hormones in Malaysian Young Adults following Helicobacter pylori Eradication
Source: PLoS One. 2015 Aug 20;10(8):e0135771. doi: 10.1371/journal.pone.0135771 (PMC4546342; doi:10.1371/journal.pone.0135771)
Supplement: S1 Table — (PDF) [file pone.0135771.s001.pdf]

Classification	^{13}C-UBT N(%)		***H. pylori* Serology N (%)**					**Overall *H. pylori* Status**	
	**Positive**	**Negative**	**Positive (IgA only)**	**Positive (IgG only)**	**Positive (IgA+IgG)**	**Seropositive**	**Seronegative**	**Positive [a]**	**Negative**
**Total**	**67 (11.7)**	**504 (88.0)**	**120**	**39**	**60**	**219 (38.2)**	**354 (61.8)**	**57 (9.9)**	**516 (90.1)**
**Ethnicity**									
Malay	12 (17.9)	225 (44.6)	46	8	10	64 (29.2)	173 (48.9)	9 (15.8)	228 (44.2)
Chinese	15 (22.4)	160 (31.8)	25	19	23	67 (30.6)	109 (30.8)	12 (21.0)	164 (31.8)
Indian	37 (55.2)	112 (22.2)	48	12	24	84 (38.4)	66 (18.6)	33 (57.9)	117 (22.7)
Other	3 (4.5)	7 (1.4)	1	0	3	4 (1.8)	6 (1.7)	3 (5.3)	7 (1.3)
**Gender**									
Male	28 (41.8)	217 (43.1)	64	12	20	96 (43.8)	149 (42.1)	22 (38.6)	223 (43.2)
Female	39 (58.2)	287 (56.9)	56	27	40	123 (56.2)	205 (57.9)	35 (61.4)	293 (56.8)
**Indeterminate**	**2 (0.3)**	**2 (0.3)**	-	-	-	-		-	-

[a]UBT with IgA and/or IgG positive were considered as *H. pylori* positive.
