# Supplementary material for: Changes in Metabolic Hormones in Malaysian Young Adults following Helicobacter pylori Eradication
Source: PLoS One. 2015 Aug 20;10(8):e0135771. doi: 10.1371/journal.pone.0135771 (PMC4546342; doi:10.1371/journal.pone.0135771)
Supplement: S2 Table — (PDF) [file pone.0135771.s002.pdf]

Histology[a]	Scale, N (%)			
	**Absent**	**Mild**	**Moderate**	**Marked**
**Neutrophils**	1 (6)	5 (31)	7 (44)	3 (19)
**Mononuclear cells**	-	-	4 (25)	12 (75)
**Atrophy**	16 (100)	-	-	-
**Intestinal metaplasia**	15 (93.8)	1 (6.2)	-	-

[a]Only sixteen RUT positive antral biopsy samples were evaluated for histology analysis.
